# No evidence for XMRV association in pediatric idiopathic diseases in France

**DOI:** 10.1186/1742-4690-7-63

**Published:** 2010-08-02

**Authors:** Eric Jeziorski, Vincent Foulongne, Catherine Ludwig, Djamel Louhaem, Gilles Chiocchia, Michel Segondy, Michel Rodière, Marc Sitbon, Valérie Courgnaud

**Affiliations:** 1 Institut de Génétique Moléculaire de Montpellier UMR 5535 CNRS, 1919 route de Mende, 34293 Montpellier cedex 5; Université Montpellier 2, Place Eugène Bataillon, 34095 Montpellier cedex 5; Université Montpellier 1, 5 Bd Henry IV, 34967 Montpellier cedex 2, France; 2 Centre Hospitalier Régional Universitaire de Montpellier, Hôpital Arnaud de Villeneuve, Service de Pédiatrie III, 371, avenue du Doyen Gaston Giraud, 34295 Montpellier cedex 5, France; 3 Centre Hospitalier Régional Universitaire de Montpellier, Hôpital Saint Eloi, Laboratoire de virologie, 80 avenue A. Fliche, 34295 Montpellier cedex 5, France; 4 Centre Hospitalier Régional Universitaire de Montpellier, Hôpital Lapeyronie, Service de chirurgie orthopédique infantile, 371, avenue du Doyen Gaston Giraud, 34295 Montpellier cedex 5, France; 5 Institut Cochin, INSERM U1016/CNRS UMR 8104, Université Paris Descartes Paris, France

## Abstract

Retroviruses have been linked to a variety of diseases such as neoplastic and immunodeficiency disorders and neurologic and respiratory diseases. Recently, a novel infectious human retrovirus, the xenotropic murine leukemia virus-related virus (XMRV), has been identified in cohorts of patients with either a familial type of prostate cancer or chronic fatigue syndrome. The apparent unrelatedness of these diseases raised the question of the potential involvement of XMRV in other diseases.

Here, we investigated the presence of XMRV in a selection of pediatric idiopathic infectious diseases with symptoms that are suggestive of a retroviral infection, as well as in children with respiratory diseases and in adult patients with spondyloarthritis (SpA). Using a XMRV *env*-nested PCR, we screened 72 DNA samples obtained from 62 children hospitalized in the Montpellier university hospital (France) for hematological, neurological or inflammatory pathologies, 80 DNA samples from nasopharyngeal aspirates from children with respiratory diseases and 19 DNA samples from SpA. None of the samples tested was positive for XMRV or MLV-like *env *sequences, indicating that XMRV is not involved in these pathologies.

## Findings

Retroviruses have been isolated from a wide variety of animal species and have been linked to a broad range of diseases, including neoplasia, non-neoplastic hematological or inflammatory diseases, immunodeficiencies and neurodegenerative and respiratory syndromes [1-3]. However in humans, it was not until the early 1980 s that two pathogenic retroviruses were isolated, a deltaretrovirus, the human T cell leukemia virus (HTLV), and a lentivirus, the human immunodeficiency virus (HIV). Both HTLV and HIV appear to have resulted from cross-species transmissions from non-human African primates involving simian T-cell leukemia viruses (STLV) and simian immunodeficiency viruses (SIV), respectively [4,5]. Interestingly, two new types of HTLV, HTLV-3 and 4 have recently been reported [6-8]. Cross-species transmission of gammaretroviruses amongst vertebrates has also been established. For example, the avian spleen necrosis virus (SNV) derives from a murine leukemia virus (MLV) and a koala endogenous retrovirus (KoRV) have been shown to be related to the gibbon ape leukemia retrovirus [9]. In 2006, an infectious human gammaretrovirus was found in prostate tissue samples from cancer patients [10]. Phylogenetic analyses revealed that this virus was closely related to several known xenotropic mouse leukemia viruses (xeno-MLV), and thus was coined XMRV for xenotropic murine leukemia virus-related virus. XMRV displays more than 90% sequence identity with MLV and harbors distinct amino acid substitutions and a short deletion in the *gag *leader region. Strikingly, these combined features lead to a putative absence of glycoGag, an alternative open reading frame of the *gag *gene that has been shown to play a role in MLV replication and pathogenesis [11]. The cellular receptor for XMRV has been shown to be the same as for xeno-MLV, i.e. XPR1 [12], a multipass membrane protein with unknown function [13]. XMRV was first described in patients who develop a familial form of prostate cancer associated with RNAse L deficiency [10]. However, in subsequent studies, a prevalence of 23% of XMRV infection in prostate cancer patients has been reported to be independent of the RNase L gene mutation [14]. More recently, XMRV has also been found, with a high prevalence, in the blood of patients with chronic fatigue syndrome (CFS), unveiling a potential broader prevalence of XMRV [15]. Most surprisingly, the prostate cancer and CFS XMRV isolates are almost identical with over 98% nucleotide sequence identity. This homology suggests that XMRV has recently arisen from a common ancestor, and that the number of replication cycles that took place during transmission and/or within one infected individual is limited.

The association of XMRV with these two pathologies remains debated in part due to the fact that several studies by European teams and a more recent one in the United States did not detect XMRV by PCR in either types of patients [16-22]. When detected, XMRV prevalence in the United States appears to be up to 40% and 67% in prostate cancer patients and CFS patients, respectively, while in Northern Europe, the prevalence is virtually zero. Furthermore, Lombardi *et al.*, found a 4% prevalence of XMRV in control patients from the same geographic region [15]. In view of the striking conservation of XMRV sequences, the lack of detection of XMRV is unlikely due to potential differences in PCR sensitivity. Therefore, differences in the worldwide distribution of XMRV may rather result from an infection that would have recently occurred in North America and that is not yet widespread in other parts of the world, or at least in Western Europe.

Retroviral pathogenesis most frequently involves hematopoietic, neurological and/or vascular symptoms through lytic, inflammatory or proliferative processes. In many human diseases of unknown etiology, retroviral involvement has recurrently been suspected. Since XMRV has been reported to be present in very different clinical entities and to a lesser extent in control samples, we wished to address the potential presence of XMRV in France, outside of CFS and prostate cancer.

While cross-species transmission is likely to take place during predatory interactions involving blood exchange, intraspecies spreading is most likely to occur through sexual exchanges or from mother-to-infant. Very few studies have been performed in pediatric samples to monitor potential retrovirus infection others than those with HIV and HTLV. In this study, we wanted to investigate XMRV as a possible etiologic agent for a selection of pediatric idiopathic diseases suggestive of retroviral infection.

Blood samples or synovial fluid cells were collected from pediatric patients less than 17 years of age admitted at the University Hospital of Montpellier (CHU Montpellier). This ongoing collection of pediatrics samples of idiopathic infectious diseases was started on September 2007, in accordance to the ethical guidelines of the French Ministry of Health (DC-2009-1052). All patients or their legal representatives have given their written informed consent.

**Figure 1:**
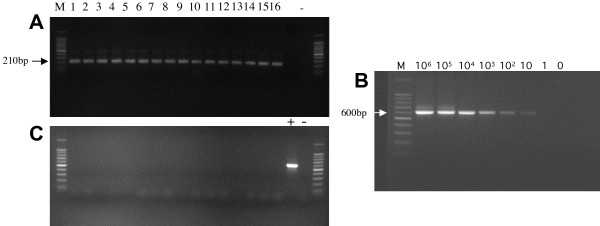
**Results of XMRV *env *nested PCR**. **(A) **GAPDH PCR on the DNA of 16 out of the 72 pediatric idiopathic diseases samples. Lanes 1-12 = DNA extracted from whole blood. Lanes 13-16 = DNA extracted from synovial fluid cells. **(B) **Sensitivity of the XMRV env PCR. Dilution series of 10^6 ^to 1 copies of a XMRV plasmid DNA in human genomic DNA. The limit of detection in our assay was 10 copies. **(C) **Nested PCR with XMRV env primers of the samples shown in A. Lane M, 100 bp marker; lane +, 600 bp PCR positive control from a XMRV env-containing plasmid; Lane-, PCR water control.

Blood samples were drawn by venipuncture using standard phlebotomy procedures into 2 ml sterile microtubes containing EDTA, and synovial fluids were obtained by needle puncture and transferred in special collection tubes. For each samples, at least 2 aliquots were prepared and stored at -80°C for later use. Total DNA was isolated from whole blood or synovial fluid cells using the QIAamp blood kit (Qiagen, Courtaboeuf, France) according to the manufacturer's instructions. DNA concentrations were determined by Nanodrop ND-1000 spectrophotometer. To ensure quality of the DNA extracts, all samples were subjected to a single-round PCR reaction using GAPDH primers (Figure 1A). Bacterial exploration with direct examination and culture was performed in all synovial fluid samples with no bacterial agent found.

The present study included 72 samples obtained from 62 children who exhibited hematological, neurological or inflammatory pathologies. All pathologies selected are listed in Table 1. In addition, we screened 80 random nasopharyngeal aspirates collected from a cohort of children aged < 5 years with respiratory diseases (including mostly bronchiolitis, >90%, pneumonia and asthma) [23].

**Table 1:** List of samples from pediatric patients.

Pediatric Pathology	Age range*	Number of patients		Sample origin
Idiopathic thrombocytopenic purpura	11 m -16 y	9	7	Whole blood
			
			1	Bone marrow
			
			1	Whole blood - Bone marrow

Autoimmune hemolytic anemia	4 y - 16 y	3	2	Whole blood
			
			1	Whole blood - Bone marrow

Aregenerative anemia	1.5 y -8 y	3	1	Whole blood
			
			1	Bone marrow
			
			1	serum

Idiopathic aplasia	12 y	1		Whole blood

Neutropenia	1 m - 3 y	4	3	Whole blood
			
			1	Bone marrow

Juvenile idiopathic arthritis	2 y -16 y	34	5	Whole blood
			
			21	Synovial fluid cells
			
			8	Whole blood - Synovial fluid cells

Henoch-Schönlein syndrome	6 y- 6 y	2		Whole blood

Encephalitis	3 y - 9 y	3		Whole blood

Dermatomyositis	9 y	1		Whole blood

Leucosis	1.5 y -15 y	2		Whole blood

* m = month, y = year

We also screened samples from 19 adult patients with spondyloarthritis (SpA), a chronic inflammatory disorder resembling the juvenile idiopathic arthritis, our largest cohort of pediatric patients. The SpA samples were previously tested for the presence of HTLV-related sequences using a sensitive semi-nested DNA amplification method allowing the detection of all PTLV-like sequences [24]. No HTLV-like sequences were found in SpA patients (unpublished data).

We designed primers to specifically target XMRV-like sequences. A 600-bp region of the SU *env *gene, spanning the receptor binding domain (RBD) was amplified with the following primers with positions indicated according to the XMRV VP35 sequence [10]: XenvS1: 5'-ATGGAAAGTCCAGCGTTCTCAAA-3' (5754 to 5776) and XenvAS1: 5'-ATGGGGACGCGGGGCCCTACATTG-3' (6443 to 6466) for the first round, while primers for the second round were XenvS2: 5'-AGGAGCCTCGGTACAACGTGACAG-3 (5840 to 5863), and XenvAS2: 5'-TGGCGGGTCAGAGAGAACAGGG-3' (6415 to 6437).

Specificity of the primers was verified in silico http://www4a.biotec.or.th/cgi-bin/webPcr and confirmed experimentally by PCR amplification on random human DNA isolated from peripheral blood mononuclear cells (PBMCs). The sensitivity of our XMRV PCR was estimated with 10-fold serial dilutions of a plasmid containing the *env *gene (kind gift from N. Fischer) in the presence of 500 ng of human PBMC DNA. In our PCR conditions, a threshold sensitivity of 10 copies per reaction was consistently achieved (Figure 1B).

Between 300 ng and 500 ng of DNA for each sample were assayed by nested PCR. PCR was performed for both rounds with High Fidelity Platinum^® ^*Taq *DNA Polymerase (Invitrogen), including a hot start (94°C for 2 min) with the following cycle conditions: 38 cycles of denaturation at 94°C for 20 s, annealing at 54°C for 30 s, and extension at 72°C for 1 min with a final elongation step at 72°C for 10 min before cooling to 4°C.

None of the 152 pediatric samples (72 various idiopathic diseases and 80 respiratory diseases) and the 19 SpA samples tested was positive for XMRV (Figure 1C) or related *env *sequence, since our primers also allowed us to detect both xeno-MLV and polytropic MLV [25,26].

In contrast with our results on pediatrics respiratory disease samples (bronchiolitis and others), Fischer *et al*. found a significant proportion of XMRV *gag *sequences in all of their respiratory disease patient and donor groups (between 2 to 10%). They found the highest incidence of *gag *XMRV detection in the group of immunosuppressed patients (adults conditioned before transplant) [27]. Although, this confirms that XMRV is more likely to emerge in the context of altered immune response, it remains perplexing that no other report found XMRV in Europe.

We showed that our nested PCR procedure is sensitive enough to detect as few as 10 copies of an XMRV *env *gene in a sample. Moreover, we have shown that we were able to detect XMRV-related *env *sequences such as xeno-MLV and the related polytropic MLV. However, we cannot formally exclude that variant viruses lacking the *env *sequences that match our primers would be present in some of these samples. Nevertheless, the remarkable conservation of XMRV *env *sequences described in all the studies published so far rather argues in favor of a bona fide absence of XMRV infection in these pathologies. Furthermore, a representative third of our samples was also unsucessfully amplified with XMRV *gag *specific primers (not shown).

As mentioned above, gammaretroviruses also participate in zoonotic transmissions [28]. Therefore, the absence of XMRV in pediatric patients as described here should not discourage the search for other gammaretroviruses potentially able to cross the species barrier through recognition of human receptors by their envelope glycoproteins.

## Abbreviations

ENV: envelope glycoprotein; GAPDH: Glyceraldehyde 3-phosphate dehydrogenase; PCR: Polymerase Chain Reaction; PTLV: Primate T-cell lymphotropic virus; SU: Env extracellular surface component.

## Competing interests

The authors declare that they have no competing interests.

## Authors' contributions

EJ was the principal experimentalist of this study who supervised sample collection and participated in the writing of the manuscript. VF performed the PCR experiments on the respiratory diseases samples and participated in the drafting of the article with MSe. LC, DJ and MR followed the patients and coordinated sample management. GC provided SpA DNA samples and participated in the drafting of the article. VC designed the experiments, coordinated their realization and initiated the manuscript writing. MSi and VC co-coordinated the realization of the study and co-wrote the manuscript. All authors read and approved the final manuscript.
